# Rhythmic Effects of Syntax Processing in Music and Language

**DOI:** 10.3389/fpsyg.2015.01762

**Published:** 2015-11-23

**Authors:** Harim Jung, Samuel Sontag, YeBin S. Park, Psyche Loui

**Affiliations:** Music, Imaging, and Neural Dynamics Lab, Psychology and Neuroscience and Behavior, Wesleyan UniversityMiddletown, CT, USA

**Keywords:** syntax, music, harmony, language, rhythm, expectancy

## Abstract

Music and language are human cognitive and neural functions that share many structural similarities. Past theories posit a sharing of neural resources between syntax processing in music and language (Patel, 2003), and a dynamic attention network that governs general temporal processing (Large and Jones, 1999). Both make predictions about music and language processing over time. Experiment 1 of this study investigates the relationship between rhythmic expectancy and musical and linguistic syntax in a reading time paradigm. Stimuli (adapted from Slevc et al., 2009) were sentences broken down into segments; each sentence segment was paired with a musical chord and presented at a fixed inter-onset interval. Linguistic syntax violations appeared in a garden-path design. During the critical region of the garden-path sentence, i.e., the particular segment in which the syntactic unexpectedness was processed, expectancy violations for language, music, and rhythm were each independently manipulated: musical expectation was manipulated by presenting out-of-key chords and rhythmic expectancy was manipulated by perturbing the fixed inter-onset interval such that the sentence segments and musical chords appeared either early or late. Reading times were recorded for each sentence segment and compared for linguistic, musical, and rhythmic expectancy. Results showed main effects of rhythmic expectancy and linguistic syntax expectancy on reading time. There was also an effect of rhythm on the interaction between musical and linguistic syntax: effects of violations in musical and linguistic syntax showed significant interaction only during rhythmically expected trials. To test the effects of our experimental design on rhythmic and linguistic expectancies, independently of musical syntax, Experiment 2 used the same experimental paradigm, but the musical factor was eliminated—linguistic stimuli were simply presented silently, and rhythmic expectancy was manipulated at the critical region. Experiment 2 replicated effects of rhythm and language, without an interaction. Together, results suggest that the interaction of music and language syntax processing depends on rhythmic expectancy, and support a merging of theories of music and language syntax processing with dynamic models of attentional entrainment.

## Introduction

Music and language are both universal human cognitive functions, but the degree to which they share cognitive resources is a long-standing debate in cognition. Theorists have argued for a shared evolutionary origin (Mithen, 2006), as well as extensive structural similarities between music and language (Lerdahl and Jackendoff, 1983; Botha, 2009), while others have argued for significant differences between music and language processing and domain specificity of the two domains (Peretz and Coltheart, 2003). Although syntax usually refers to the rules that govern how words and phrases are arranged in language, syntactic structure also exists in other domains, such as music. Musical syntax can be understood as the rules that define how pitches are organized to form melody and harmony. Western tonal harmony, like language, is organized in hierarchal structures that are built upon discrete and combined elements (Lerdahl and Jackendoff, 1983). Syntax in Western music can be realized in the structured organization of the 12 chromatic tones into diatonic scale degrees within tonal centers, which form chords within harmonic progressions. Both musical and linguistic structures unfold syntactically over time.

One theory that has influenced research in the structures of music and language is the Shared Syntactic Integration Resource Hypothesis (SSIRH), which postulates an “overlap in the neural areas and operations which provide the resources for syntactic integration” (Patel, 2003). The hypothesis reconciles contrasting findings between neuropsychology and neuroimaging studies on syntax processing, by suggesting that the same syntactic processing mechanisms act on both linguistic and musical syntax representations. The SSIRH predicts that the syntactic processing resources are limited, and thus studies with tasks combining musical and linguistic syntactic integration will show patterns of neural interference (Patel, 2003). While topics of ongoing debate concern the nature of the resources that are shared (Slevc and Okada, 2015) and the extent to which such resources are syntax-specific (Perruchet and Poulin-Charronnat, 2013), convergent studies do provide evidence for some shared processing of music and language, with evidence ranging from behavioral manipulations of syntactic expectancy violations in music and language (e.g., Fedorenko et al., 2009; Slevc et al., 2009; Hoch et al., 2011) to cognitive neuroscience methods such as ERP and EEG studies that track the neural processing of syntax and its violations (e.g., Koelsch et al., 2005; Steinbeis and Koelsch, 2008; Fitzroy and Sanders, 2012).

One piece of evidence in support of the shared processing of musical and linguistic syntax comes from a reading time study in which musical and linguistic syntax were manipulated simultaneously (Slevc et al., 2009). Reading time data for a self-paced reading paradigm showed interactive effects when linguistic and musical syntax were simultaneously violated, suggesting the use of the same neural resources for linguistic and musical syntax processing. In this self-paced reading paradigm, linguistic syntax was violated using garden path sentences, whereas musical syntax was violated using harmonically unexpected musical chord progressions.

As both musical and linguistic syntax unfold over time, the timing of both musical and linguistic events may affect such sharing of their processing resources. Rhythm, defined as the pattern of time intervals in a stimulus sequence, is usually perceived as the time between event onsets (Grahn, 2012a). As a pattern of durations that engenders expectancies, rhythm may represent its own form of syntax and thus be processed similarly to both musical and linguistic syntax in the brain (Fitch, 2013). It has also been suggested that rhythm is an implicitly processed feature of environmental events that affects attention and entrainment to events in various other domains such as music and language (Large and Jones, 1999). Specifically, the Dynamic Attending Theory (DAT) posits a mechanism by which internal neural oscillations, or attending rhythms, synchronize to external rhythms (Large and Jones, 1999). In this entrainment model, rhythmic processing is seen as a fluid process in which attention is involuntarily entrained, in a periodic manner, to a dynamically oscillating array of external rhythms, with attention peaking with stimuli that respect the regularity of a given oscillator (Large and Jones, 1999; Grahn, 2012a). This process of rhythmic entrainment has been suggested to occur via neural resonance, where neurons form a circuit that is periodically aligned with the stimuli, allowing for hierarchical organization of stimuli with multiple neural circuits resonating at different levels, or subdivisions, of the rhythm (Large and Snyder, 2009; Grahn, 2012a; Henry et al., 2015). One piece of evidence in support of the DAT comes from Jones et al. (2002), in which a comparative pitch judgment task was presented with interleaving tones that were separated temporally by regular inter-onset intervals (IOIs) that set up a rhythmic expectancy. Pitch judgments were found to be more accurate when the tone to be judged was separated rhythmically from the interleaving tones by a predictable IOI, compared to an early or late tone that was separated by a shorter or longer IOI, respectively. The temporal expectancy effects from this experiment provide support for rhythmic entrainment of attention within a stimulus sequence.

Both SSIRH and DAT make predictions about how our cognitive system processes events as they unfold within a stimulus sequence, but predictions from SSIRH pertain to expectations for linguistic and musical structure, whereas those from DAT pertain to expectations for temporal structure. The two theories should converge in cases where expectations for music, language, and rhythm unfold simultaneously.

### Aims and overall predictions

The current study aims to examine the simultaneous cognitive processing of musical, linguistic, and rhythmic expectancies. We extend the reading time paradigm of Slevc et al. (2009), by borrowing from the rhythmic expectancy manipulations of Jones et al. (2002), to investigate how the introduction of rhythmic expectancy affects musical and linguistic syntax processing. Rhythmic expectancy was manipulated through rhythmically early, on-time, or late conditions relative to a fixed, expected onset time. As previous ERP data that have shown effects of temporal regularity in linguistic syntax processing (Schmidt-Kassow and Kotz, 2008), it is expected that rhythmic expectancy does affect syntax processing. The current behavioral study more specifically assesses how rhythmic expectancy may differentially modulate the processing of musical and linguistic syntax.

## Experiment 1

### Methods

Participants read sentences that were broken down into segments, each of which was paired with a chord from a harmonic chord progression. Linguistic syntax expectancy was manipulated using syntactic garden-path sentences, musical expectancy was manipulated using chords that were either in key or out of key, and rhythmic expectancy was manipulated by presenting critical region segments early, on time, or late.

#### Participants

Fifty six undergraduate students from Wesleyan University participated in this study in return for course credit. A recording error resulted in the loss of data for 8 out of the 56 total students, and so 48 participants' data were used in the final analysis. Of the remaining participants, all reported normal hearing. Twenty eight participants (58.3%) reported having prior music training, averaging 6.8 years (*SD* = 3.4). Twenty five (52%) participants identified as female, and 23 as male. Thirty eight (79.1%) reported that their first language was English, three were native speakers of English and one other language, and seven had a language other than English as their first language. Other than English, participants' first languages included Chinese (Mandarin), Arabic, Thai, Japanese, Spanish, French, German, Vietnamese, and Bengali. Sixteen participants (33.3%) spoke more than one language. All participants had normal or corrected-to-normal vision and reported being free of psychiatric or neurological disorders. Informed consent was obtained from all subjects as approved by the Ethics Board of Psychology at Wesleyan University.

#### Materials

All experiments were conducted in Judd Hall of Wesleyan University. An Apple iMac and Sennheiser HD280 pro headphones were used for the experiments, with MaxMSP software (Zicarelli, 1998) for all stimulus presentation and response collection.

#### Stimuli

**Figure 1:**
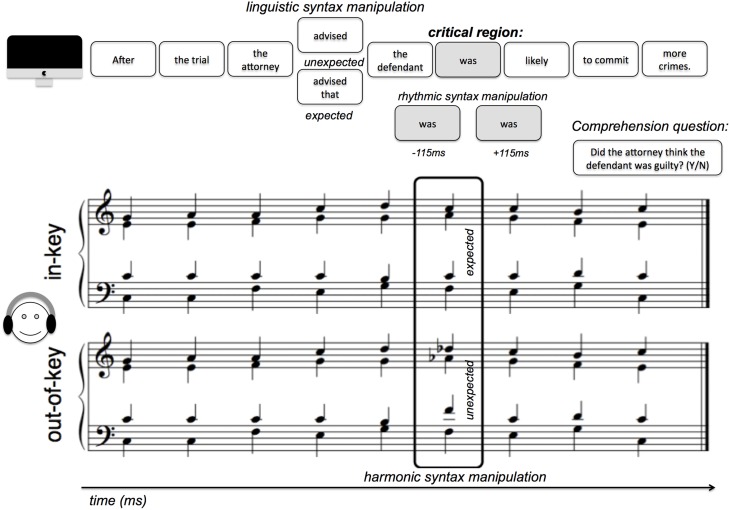
**Experiment design: Schematic illustration of experimental design and stimuli presented in one trial**.

**Figure 2:**
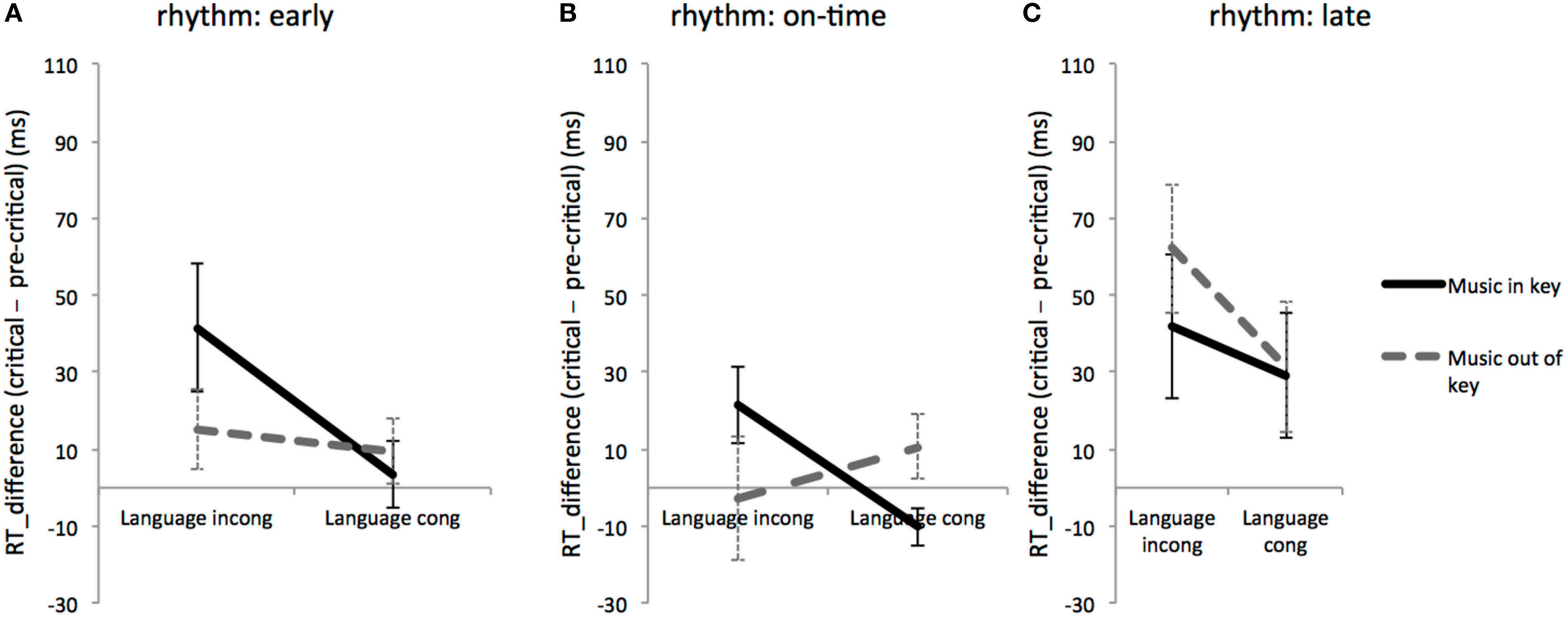
**Rhythmic effects on music and language: RT differences between critical region and pre-critical region for linguistically and musically expected and unexpected conditions during rhythmically early (A), on-time (B), and late (C) conditions**. Error bars show standard error.

The current study used 48 sentences from Slevc et al. (2009). These sentences were divided into segments of one or several words, and presented sequentially on the iMac screen using MaxMSP. Twelve of the sentences were syntactic garden paths, which were manipulated to be either syntactically expected or unexpected at the critical region (by introducing a garden path effect—see **Figure 2**). Reading time (RT) comparisons between different conditions were controlled for length of segment because the critical regions are always the same number of words (as shown in Figure 1) in the different conditions. Sentence segments with the paired harmonic progression were presented at a critical region, either on-time (at the regular inter-onset interval of 1200 ms) or “jittered” to be either early or late. The early jitter was 115 ms earlier than the on-time presentation, and the late jitter was 115 ms later than the on-time presentation. Thus, the IOIs were either 1200–115 = 1085 ms (early), 1200 ms (on-time), or 1200+115 = 1315 ms (late; Figure 2). 115 ms was selected as the temporal jitter based on pilot testing and the IOIs used in Experiment 2 of Jones et al. (2002) in their manipulation of temporal expectancy. Accompanying chord progressions were played in MIDI using a grand piano timbre. These 48 different progressions were also from Slevc et al. (2009) and followed the rules of Western tonal harmony, and were all in the key of C major. Out-of-key chords violated harmonic expectancy given the context, but were not dissonant chords by themselves (Figure 1). A yes-or-no comprehension question was presented at the end of each trial (sentence). Participants' task was to press the spacebar on the keyboard as soon as they had read each sentence segment, and to answer “yes” or “no” to the comprehension questions. Ninety six unique comprehension questions, two for each sentence, were written so each sentence would have one comprehension question written to have a correct answer “yes,” and another to have a correct answer “no.” The comprehension questions are now given in the Supplementary Materials accompanying this manuscript.

Twelve unique experimental modules were created in order to counterbalance the experimental design. Each module contained all 48 sentences, with violation and filler conditions rotated through the sentences in order to control for systematic effects of content, length, and sentence order. Each module contained: 4 rhythmic violation trials (2 early and 2 late), 3 musical syntax violation trials, 1 linguistic syntax violation trial, 5 musical syntax plus rhythmic violation trials, 1 linguistic plus musical syntax violation trial, 2 linguistic syntax plus rhythmic violation trial, 2 trials with all 3 violations, and 30 sentences with no violations. Therefore, in a given module only 37.5% of trials contained any violation. Half of the sentences in a given module were assigned a “yes” question, the other half were assigned a “no.” The order of the trials was randomized for each subject.

#### Procedure

Before beginning the experiment, the participants gave informed consent and completed a short background survey. The participants were then instructed to pay close attention to the sentences being read, rather than the chord progressions that were heard over the headphones. Then, the participants ran through a set of practice trials. After the practice trials, in the actual experiment the experimenter selected one of the 12 possible experimental modules at random. Participants were instructed to press the spacebar on the keyboard as soon as they had read the sentence segment, and then wait for the next segment to be presented. Pressing the spacebar caused the current sentence segment to disappear and an indicator button labeled “I read it” to light up. The following segment appeared at a fixed IOI regardless of when the current segment disappeared. After the end of each sentence, a yes-or-no comprehension question was displayed, at which point participants answered the question by pressing Y or N on the keyboard. Answering the comprehension question cued a new trial. The experiment lasted ~20 min. Examples of different types of trials are shown in a video demo in the Supplementary Materials accompanying this manuscript.

#### Data analysis

RT and response data were saved as text files from MaxMSP, and imported into Microsoft Excel and SPSS for statistical analysis. RTs were log-transformed to normal distribution for statistical testing. Only RTs pre-critical, critical, and post-critical regions for each trial were used for analysis. Filler trials were, therefore, excluded from analysis (21 trials per subject). Of the remaining trials, trials with RTs that were two or more standard deviations from the mean of log-transformed critical region RTs were excluded as outliers, resulting in a range of 102.76–816.74 ms. These criteria led to the exclusion of 92 (7.20%) of observations from critical regions in Experiment 1.

No significant differences were observed in log-transformed RTs between native English speakers (*n* = 41) and non-native English speakers [non-native *n* = 7, *t*_(46)_ = 0.42, n.s.]. Similarly, no significant differences were observed between participants who reported musical training (*n* = 29) and those who reported no musical training [*n* = 19, *t*_(46)_ = 1.53, n.s.]. To check for interactions between linguistic syntax and native English speaker experience, an ANOVA was run on the dependent variable of log-transformed RT with the fixed factor of linguistic syntax (congruent vs. incongruent) and the random factor of native English speaker status (native vs. non-native English speaker). No significant interaction between native English speaker status and linguistic syntax was observed [*F*_(1, 92)_ = 0.53, *MSE* = 0.01, *p* = 0.47]. Similarly, to check for interactions between musical syntax and musical training, an ANOVA with the fixed factor of musical syntax (congruent vs. incongruent) and the random factor of musical training (musically trained vs. no musical training) showed no interaction between musical syntax and musical training [*F*_(1, 92)_ = 0.091, *MSE* = 0.008, *p* = 0.764]. As we observed no main effects or interactions that were explainable by native English speaking experience or musical training, results were pooled between native and non-native English speakers, and between musically trained and untrained subjects.

### Results

On comprehension questions, participants performed significantly above chance in all conditions [overall *M* = 78.95%, *s* = 12.24, two-tailed *t*-test against chance level of 50% correct: *t*_(47)_ = 16.38, p < 0.0001].

A Three-way ANOVA on the dependent variable of log-transformed RT during the critical region (log_RT_CR) was run with fixed factors of language (two levels: congruent and incongruent), music (two levels: congruent vs. incongruent), and rhythm (three levels: early, on-time, and late), with subject number as a random factor. Results showed a significant three-way interaction among the factors of linguistic, musical and rhythmic expectancies [*F*_(2, 52)_ = 5.02, *MSE* = 0.008, *p* = 0.01], as well as a significant main effect of language [*F*_(1, 54)_ = 12.5, *MSE* = 0.006, *p* = 0.001] and a significant main effect of rhythm [*F*_(2, 99)_ = 13.2, *MSE* = 0.01 *p* < 0.001] and a marginally significant effect of music [*F*_(1, 53)_ = 3.7, *MSE* = 0.01, *p* = 0.059]. Means and SDs of RTs are given in Table 1 for each condition, and in Table 2 for each cell.

**Table 1:** **Mean critical region RTs (ms) under different conditions of linguistic syntax, musical syntax, and rhythmic expectancies**.

	**Lang**			**Music**			**Rhythm**	
	***M***	***SD***		***M***	***SD***		***M***	***SD***
Congruent	311.8	63.15	In-key	315.91	65.85	Early	327.1	80.62
Incongruent	339.12	84.81	Out-of-key	322.67	69.78	On-Time	301.12	67.92
						Late	351.6	71.24

**Table 2:** **Mean critical region RTs (ms) under different combinations of conditions of linguistic syntax, musical syntax, and rhythmic expectancies**.

	**Early**				**On time**				**Late**			
	**Music**				**Music**				**Music**			
	**In key**		**Out key**		**In key**		**Out key**		**In key**		**Out key**	
**Language**	***M***	***SD***	***M***	***SD***	***M***	***SD***	***M***	***SD***	***M***	***SD***	***M***	***SD***
Congruent	326.22	100.59	313.81	88.72	294.53	65.86	307.84	106.92	369	101.44	334.62	89.4
Incongruent	361.72	126.53	316.09	76.31	331.16	102.5	310.03	116.35	365.37	203.76	388.32	139.02

To investigate any possible interactive effects between music and language syntax at different rhythmic conditions, an RT difference was computed between RTs for critical region and for pre-critical region. Two-way ANOVAs with fixed factors of language and music were used to test for interactions between music and language at each of the three rhythm conditions (early, on-time, and late). Results showed that for the rhythmically on-time condition, there was an interaction between language and music [*F*_(1, 170)_ = 4.9, *MSE* = 4776.9, *p* = 0.027]. In contrast, the interaction between language and music was not significant at the rhythmically early condition [*F*_(1, 170)_ = 0.27, *MSE* = 12882.0, *p* = 0.603] or the rhythmically late condition [*F*_(1, 170)_ = 2.34, *MSE* = 5155.2, *p* = 0.127] (see Figure 2). These results suggest that the interaction between linguistic and musical syntax varies by rhythmic expectancy.

**Figure 3:**
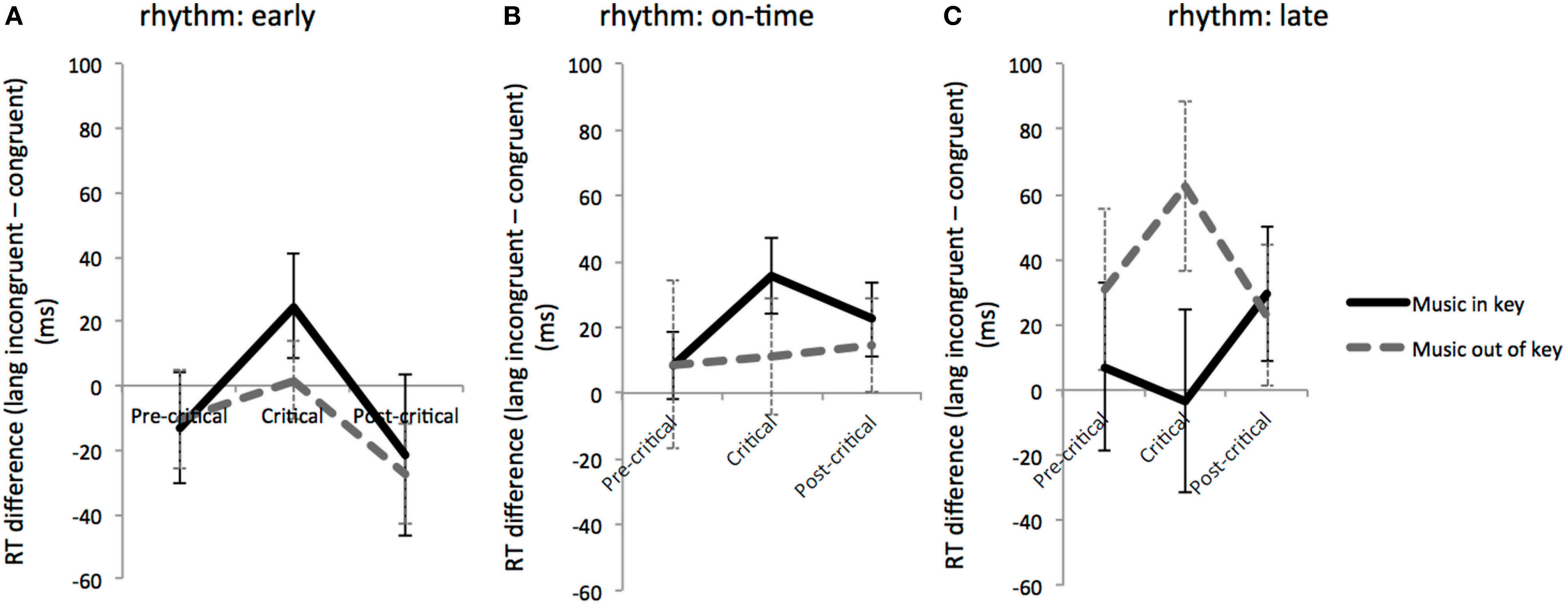
**Reading time differences: RT differences between linguistically congruent and incongruent conditions for musically expected and unexpected conditions at different time windows (pre-critical, critical, and post-critical) during rhythmically early (A), on-time (B), and late (C) conditions**. Error bars show standard error.

Further investigation of the degree to which factors interacted at the critical region required comparing RTs across the pre-critical, critical, and post-critical time regions. For this comparison, difference scores of linguistically congruent from linguistically incongruent RTs were calculated, and these difference scores were compared for musically in-key and out-of-key trials across time regions for each rhythmic condition (see Figure 3). We found a significant effect of time region: RT was longer in the critical region in the rhythmically early condition only [*F*_(2, 92)_ = 4.67, *p* = 0.012]. In the rhythmically late condition only, musical syntax violations produced larger difference scores at the critical region; however this difference was not significant. In the rhythmically early condition and on-time conditions, musically in-key trials yielded larger difference scores than musically out-of-key trials at the critical regions, although these differences were not significant (see Figure 3).

### Discussion

Experiment 1 tested to see how rhythmic expectancy affected the processing of musical and linguistic syntax. Results from log-transformed RTs during the critical region (Table 2) and RT differences between critical and pre-critical regions (Figure 2) showed significant main effects of language and rhythm, a significant three-way interaction of language, music, and rhythm, and a significant two-way interaction between linguistic and musical syntax in the on-time condition only. These findings extend the results of past research (Slevc et al., 2009) to show that the sharing of cognitive resources for music and language appear specific to rhythmically expected events.

In contrast to critical region RTs, however, RT differences between linguistically incongruent and congruent trials (Figure 3) showed slower RTs within the critical region only during rhythmically early trials. The interaction patterns between musical and linguistic syntax over different time regions were inconclusive. This differs from the original findings of Slevc et al. (2009), who observed a synergistic interaction between musical syntax and time region on the reaction time difference between linguistically congruent minus incongruent trials, suggestive of a language and music interaction specifically during the critical region, when rhythm was not a factor. The less robust effect of critical region in this experiment may arise from spillover effects of linguistic incongruence that last beyond the critical region.

While neither SSIRH nor DAT makes specific predictions about this possible spillover effect, the main findings of a three-way interaction among language, music, and rhythm is generally consistent with both theoretical accounts and does suggest that any synergy or sharing of neural resources between music and language depends on rhythmic expectancy. Violations in rhythmic expectancy may disrupt the shared resources that are generally recruited for syntax processing, such as cognitive control (Slevc and Okada, 2015). As music and language both unfold over time, it stands to reason that our expectations for rhythm—defined here as the pattern of time intervals within a stimulus sequence (Grahn, 2012a)—would govern any sharing of neural resources between music and language, as is consistent with the DAT (Large and Jones, 1999), as well as prior behavioral data on rhythmic entrainment (Jones et al., 2002) and studies on the neural underpinnings of rhythmic entrainment (Henry et al., 2015) and their effects on linguistic syntax processing (Schmidt-Kassow and Kotz, 2008).

The three-way interaction between language, music, and rhythm is accompanied by significant main effects of language and rhythm, and marginally significant main effect of musical expectancy. The main effect of rhythm is similar to Jones et al. (2002) and others, in which perturbed temporal expectations resulted in longer RTs. Incongruent garden-path sentences elicit longer RTs during the critical region compared to their counterparts. This is consistent with Slevc et al. (2009) and Perruchet and Poulin-Charronnat, 2013) as well as with previous uses of the self-paced reading time paradigm (Ferreira and Henderson, 1990). The main effect of musical expectancy was only marginally significant. While it is worth noting that Slevc et al. (2009) also did not report a significant main effect of musical expectancy, this weak effect may also be due to task instructions to pay close attention to the sentence segments rather than to the chord progressions heard over headphones. To determine whether music generally taxed cognitive or attentional resources away from subjects' monitoring of the sentence segments, it was necessary to compare comprehension accuracy with and without musical stimuli. This was a motivation for Experiment 2, in which the experiment was re-run without musical stimuli.

While previous studies that used a self-paced reading paradigm (Ferreira and Henderson, 1990; Trueswell et al., 1993; Slevc et al., 2009; Perruchet and Poulin-Charronnat, 2013) required subjects to activate the next sentence segment as part of the task, in order to implement a factor of rhythmic expectancy our design featured a fixed inter-onset interval of sentence segments, and subjects were asked instead to press a button to indicate that they had read each segment. To our knowledge this type of implementation is new for psycholinguistic studies. One of the goals of Experiment 2 is to check for the validity of this type of implementation by testing for an effect of linguistic congruency with fixed IOI presentations of sentence segments, even in the absence of musical stimuli.

## Experiment 2

Our modification of the standard self-paced reading paradigm resulted in fixed IOIs with the task of indicating that subjects had read the displayed sentence segment. This was a different task from the standard self-paced reading paradigm in which subjects' task was to advance the following sentence segment, and our task had yet to be confirmed as effective in detecting effects of linguistic syntax, even without the presence of musical stimuli. Furthermore, it was possible that the three-way and two-way interactions from Experiment 1 resulted from the complexity of our experimental design, and that the processing of multiple violations could affect attending and development of expectancy to task-irrelevant stimuli, as well as syntax processing *per se*. Experiment 2 thus follows up on Experiment 1 by investigating effects of rhythmic violations on comprehension and the processing of linguistic syntax stimuli, removing the variable of musical stimuli. A significant effect of linguistic syntax as well as rhythmic expectancy could validate the current manipulation of the self-paced reading paradigm, and a significant interaction between language and rhythm would suggest that the two domains tap into the same specific neural resources whereas no interaction might suggest more parallel processing.

### Methods

In experiment 2, participants again read sentences broken down into segments. Linguistic syntax expectancy was manipulated using syntactic garden-path sentences, and rhythmic expectancy was manipulated by presenting critical region segments early, on-time, or late.

#### Participants

A new group of 35 undergraduate students from Wesleyan University participated in Experiment 2 in return for course credit. From these participants, all reported normal hearing, normal or corrected-to-normal vision, and no psychiatric or neurological disorders. Twenty-five participants (71.4%) reported having prior music training, averaging 5.9 years (*SD* = 3.0). Twenty (57.1%) participants identified as female, and 15 (42.3%) as male. Twenty-eight (80%) reported that their first language was English, and seven had a language other than English as their first language. Other than English, participants' first languages included Spanish, Chinese, and Thai. Twenty-four participants (68.6%) spoke more than one language. Informed consent was obtained from all subjects as approved by the Ethics Board of Psychology at Wesleyan University.

#### Materials

The second experiment was conducted in the Music, Imaging, and Neural Dynamics (MIND) Lab Suite in Judd Hall at Wesleyan University. An Apple iMac was used for the experiment, with MaxMSP software for all stimulus presentation and response collection.

#### Stimuli

The same experimental patch on MaxMSP and 12 experimental modules with the 48 sentences borrowed from Slevc et al. (2009) were used from the first experiment. However, to investigate how rhythmic violations would affect reading and interact with violations in linguistic syntax, independent of violations in musical syntax, the experimental patch was muted, so that chords were not heard with each sentence segment. The IOIs of sentence segments remained unaltered, and the same “yes” or “no” comprehension questions were also asked at the end of each trial, with randomized order of the trials for each subject.

#### Procedure

Similar to Experiment 1, participants were instructed to read sentences carefully, and hit the spacebar as soon as they had read a sentence segment. After running through a practice set, the participants began the actual experiment. The experimenter selected one of the twelve possible experimental modules at random. At the end of each trial, participants answered the “yes” or “no” comprehension question, queuing the next trial.

#### Data analysis

RTs and comprehension question responses were saved as text files from MaxMSP, and imported into Microsoft Excel, and SPSS for statistical analysis. Only RTs at the pre-critical, critical, and post-critical regions for each trial were used for analysis. Filler trials were, again, excluded from analysis (21 trials per subject). The same parameters and methods of outlier exclusion were used from the previous experiment, resulting in a RT range of 123.63–1121.40 ms. These criteria led to the exclusion of 19 (1.97%) of observations in Experiment 2. RTs were also log-transformed to normal distribution for statistical tests.

Results between musically trained and non-musically trained subjects were pooled because music was not a factor in this experiment. No significant differences were observed in log-transformed RTs between native English speakers and non-native English speakers [*t*_(34)_ = 0.96, n.s.]. Similarly, an ANOVA with the fixed factor of linguistic syntax and the random factor of native English experience showed no significant interaction [*F*_(1, 523)_ = 1.059, *MSE* = 0.018, *p* = 0.30]. As we observed no differences that were explainable by native English speaking experience, results were pooled between native and non-native English speakers.

### Results

Participants performed significantly above chance (*M* = 86.93%, *s* = 6.21) on comprehension questions in all conditions. To compare comprehension accuracy with and without musical stimulus presentation, a One-way ANOVA on average comprehension accuracy as the dependent variable was run with the factor of experiment, comparing average comprehension accuracy for subjects between Experiment 1 and 2. Results showed a significant main effect of experiment on comprehension accuracy, with subjects from Experiment 2 performing better on average on comprehension questions than those from Experiment 1 [*F*_(1, 81)_ = 12.51, *MSE* = 0.01, *p* = 0.001]. This suggests that the added variable of musical expectancy further taxed participants' attention from the task-relevant comprehension questions in Experiment 1.

A Two-way ANOVA on the dependent variable of log-transformed RT during the critical region was run with the factors of language and rhythm. Results showed a significant main effect of language [*F*_(1, 34)_ = 7.69, *MSE* = 0.001. *p* = 0.009], a significant effect of rhythm [*F*_(2, 68)_ = 9.69, *MSE* = 0.001, *p* < 0.001], and no significant two-way interaction [*F*_(2, 68)_ = 1.07, *MSE* = 0.001, *p* = 0.83]. Mean and SD RTs are shown for each condition in Table 3 and for each cell in Table 4.

**Table 3:** **Mean critical region RTs (ms) under different conditions of linguistic and rhythmic expectancies**.

	**Lang**			**Rhythm**	
	***M***	***SD***		***M***	***SD***
Congruent	387.34	57.2	Early	415.26	64.21
Incongruent	414.13	87.64	On-Time	381.39	62.18
			Late	399.11	75.97

**Table 4:** **Mean critical region RTs (ms) under different combinations of conditions of linguistic syntax and rhythmic expectancies**.

	**Early**		**On time**		**Late**	
**Language**	***M***	***SD***	***M***	***SD***	***M***	***SD***
Congruent	407.41	68.06	377.65	59.45	398.16	82.17
Incongruent	434.84	116.69	397.2	109.48	412.56	125.85

### Discussion

Results from Experiment 2 showed main effects of language and rhythm, validating the use of this novel task. There was also a higher comprehension accuracy compared to Experiment 1, but no interactions between the two factors of linguistic syntax and rhythmic expectancy (see Table 4).

Experiment 2 further investigates the effects of rhythmic expectancy on linguistic syntax processing. When the factor of music was removed, main effects of language and rhythm were still observed. RTs were longer for syntactically unexpected sentences, replicating results from Experiment 1 as well as previous experiments that used the self-paced reading time paradigm (Ferreira and Henderson, 1990; Trueswell et al., 1993). Notably, this finding of longer RTs during syntactically unexpected critical regions within the garden path sentences provides a validation of the current adaptation of the self-paced reading time paradigm: while previous studies that used the self-paced reading time paradigm (Ferreira and Henderson, 1990; Trueswell et al., 1993; Slevc et al., 2009; Perruchet and Poulin-Charronnat, 2013) required subjects to advance the sentence segments manually, in the current study we adapted the paradigm with fixed IOIs to enable simultaneous investigations of rhythmic and linguistic syntax expectancy.

Effects of rhythmic expectancy were also observed, as participants were slower to respond to critical regions presented earlier or later than the expected IOI. This replicates results from Experiment 1 and suggests that temporal entrainment was possible even with a visual-only reading task, and thus is not limited to the auditory modality. This effect of rhythm on visual processing is consistent with prior work on rhythmic effects of visual detection (Landau and Fries, 2012) and visual discrimination (Grahn, 2012b).

Although main effects of language and rhythm were observed, there was no significant interaction. An explanation for this lack of interaction could be that removing the factor of music resulted in the implemented violations no longer being sufficiently attention-demanding to lead to an interaction between the remaining factors, resulting in parallel processing of language and rhythm. In this view, the data suggests that rhythm affects a general, rather than a syntax-specific, pool of attentional resources. When the factor of music was removed, attentional resources were less demanded from the available pool, reducing the interactive effects of language and rhythm on each other and resulting in no interaction and higher comprehension accuracy. Alternately, it could be that the rhythm only affected peripheral visual processing, without also affecting syntax processing at a central level. While the present experiment cannot tease apart these possible explanations, considering the extant literature on relationships between rhythm and grammar (Schmidt-Kassow and Kotz, 2009; Gordon et al., 2015b) it is clear that rhythm can affect central cognitive processes such as syntactical or grammatical computations.

Finally, another finding from Experiment 2 is that comprehension accuracy was higher compared to Experiment 1, suggesting that eliminating the factor of music restored some attentional resources to the task of comprehension. When the primary task was to read sentence segments for comprehension, musical stimuli in the background could have functioned as a distractor in a seeming dual-task condition of comprehending the entire sentence while responding to each segment (by pressing the spacebar).

Taken together, Experiment 2 helps to validate the paradigm used in Experiment 1. By simplifying the experiment to remove the factor of music, some attentional resources may have been restored, resulting in higher comprehension accuracy overall, as well as main effects of language and rhythm with no interaction between the two.

## General discussion

The goal of the current study is to examine how rhythmic expectancy affects the processing of musical and linguistic syntax. Experiment 1 shows main effects of language, music, and rhythm, and specificity of the interaction between musical and linguistic syntax in the rhythmically expected condition only. These data patterns confirm that rhythm affects the sharing of cognitive resources for music and language, and is largely consistent with SSIRH (Patel, 2003) and DAT (Large and Jones, 1999). However, some of the follow-up analyses are inconclusive as to the exact nature of these interactions over time. In particular, only in rhythmically early trials did we find that the critical region significantly affected the difference in RT between incongruent and congruent language trials, with no significant interactions with musical expectancy unlike in Slevc et al. (2009). The reason for this specific effect of critical region in rhythmically early trials is unclear. It might arise from some spillover effects of linguistic incongruence that last beyond the critical region in rhythmically on-time and late trials. Alternately, it might be a consequence of the complexity of our task in this experiment design. Although the significant main effects suggest that our manipulations were effective, this inconclusive data pattern may nevertheless result from low power due to relatively few trials per cell in the experiment design of Experiment 1.

As it is possible that results were due to the complexity of our design, Experiment 2 simplifies the design by eliminating the factor of music altogether. Results of Experiment 2 show superior comprehension accuracy compared to Experiment 1, and main effects of language and rhythm without an interaction between the two factors. The main effects help to validate our adaptation of the original self-paced reading time paradigm (Ferreira and Henderson, 1990; Trueswell et al., 1993) for research in rhythmic expectancy. The null interaction, when accompanied by significant main effects, suggests that given the task conditions and attentional allocation in Experiment 2, rhythm and language were processed in parallel and did not affect each other.

The superior comprehension accuracy in Experiment 2 may be explained by an increase in general attentional resources that are now available to subjects in Experiment 2 due to the removal of music as a factor. While it was not specifically tested whether these general attentional mechanisms may be the same or different from the temporal attention that is taxed by temporal perturbations of rhythmic expectancy, other literature on voluntary (endogenous) vs. involuntary (exogenous) attention might shed light on this distinction (Hafter et al., 2008; Prinzmetal et al., 2009). Voluntary or endogenous attention, such as that tested in dual-task situations when the task is to attend to one task while ignoring another, is similar to the general design of the present studies where subjects are instructed to pay attention to sentence segments while ignoring music that appears simultaneously. Involuntary or exogenous attention, in contrast, is driven by stimulus features such as rhythmic properties as tapped by our rhythmic expectancy manipulations. Previous research has shown that voluntary attention tends to affect accuracy whereas involuntary attention affects reaction time (Prinzmetal et al., 2005). This fits with our current findings where comprehension accuracy is affected by the removal of music as a factor (by comparing Experiments 1 and 2), whereas reading time is affected by rhythmic perturbations of the presentation of sentence segments.

In both experiments, effects of rhythm were observed in response to visually-presented sentence segments. While the rhythmic aspect of language might generally manifest itself more readily in the auditory than the visual modality, this effect observed from the visual manipulations suggests that rhythmic expectation for language is not limited to auditory processing, but may instead pervade the cognitive system in a modality-general manner, affecting even the visual modality. As visual detection and discrimination are both modulated by rhythm (Grahn, 2012b; Landau and Fries, 2012) and musical expectation can cross-modally affect visual processing (Escoffier and Tillmann, 2008), the current study provides support for the view that rhythmic, musical, and linguistic expectations are most likely not tied to the auditory modality, but instead affect the cognitive system more centrally.

Results appear to be independent of musical training and native English speaker experience. The link between linguistic and musical grammar processing could have been expected to vary by musical and linguistic expertise: children who perform well on phonemic or phonological tasks also outperform their counterparts in rhythmic discrimination as well as pitch awareness (Loui et al., 2011; Gordon et al., 2015b). At a neural level, brain areas and connections that subserve language are different in their structure and function among professional musicians (Sluming et al., 2002; Halwani et al., 2011), and some highly trained populations, such as jazz drummers, process rhythmic patterns in the supramarginal gyrus, a region of the brain that is thought to be involved in linguistic syntax (Herdener et al., 2014). Despite these effects of training and expertise, the current study found no effects of musical training or linguistic background, converging with the original study (Slevc et al., 2009) as well as prior reports of the language-like statistical learning of musical structure (Loui et al., 2010; Rohrmeier et al., 2011). It is possible that only some types of task performance, such as those that tap more sensory or perceptual resources, might be affected by music training via selective enhancement of auditory skills (Kraus and Chandrasekaran, 2010).

In sum, the current study demonstrates that rhythmic expectancy plays an important role in the shared processing of musical and linguistic structure. The subject of shared processing of musical and language structure has been central to music cognition, as is the question of how rhythm affects attentional entrainment. While providing support for an overlap in processing resources for musical and linguistic syntax, the current results also suggest that perturbations in rhythmicity of stimuli presentation tax these attentional resources. By offering a window into how perturbations of rhythmic and temporal expectancy affect musical and linguistic processing, results may be translatable toward better understanding and possibly designing interventions for populations with speech and language difficulties, such as children with atypical language development (Przybylski et al., 2013; Gordon et al., 2015a). Toward that goal, the specific neural underpinnings of these shared processing resources still remain to be addressed in future studies.

### Conflict of interest statement

The authors declare that the research was conducted in the absence of any commercial or financial relationships that could be construed as a potential conflict of interest.

## References

[B1] Botha R. (2009). On musilanguage/“Hmmmmm” as an evolutionary precursor to language. Lang. Commun. 29, 61–76. 10.1016/j.langcom.2008.01.001

[B2] Escoffier N. Tillmann B. (2008). The tonal function of a task-irrelevant chord modulates speed of visual processing. Cognition 107, 1070–1083. 10.1016/j.cognition.2007.10.007 18076873

[B3] Fedorenko E. Patel A. Casasanto D. Winawer J. Gibson E. (2009). Structural integration in language and music: evidence for a shared system. Mem. Cognit. 37, 1–9. 10.3758/MC.37.1.1 19103970

[B4] Ferreira F. Henderson J. M. (1990). Use of verb information in syntactic parsing: evidence from eye movements and word-by-word self-paced reading. J. Exp. Psychol. Learn. Mem. Cogn. 16, 555–568. 10.1037/0278-7393.16.4.555 2142952

[B5] Fitch W. T. (2013). Rhythmic cognition in humans and animals: distinguishing meter and pulse perception. Front. Syst. Neurosci. 7:68. 10.3389/fnsys.2013.00068 24198765 PMC3813894

[B6] Fitzroy A. B. Sanders L. D. (2012). Musical expertise modulates early processing of syntactic violations in language. Front. Psychol. 3:603. 10.3389/fpsyg.2012.00603 23335905 PMC3542524

[B7] Gordon R. L. Jacobs M. S. Schuele C. M. McAuley J. D. (2015a). Perspectives on the rhythm-grammar link and its implications for typical and atypical language development. Ann. N.Y. Acad. Sci. 1337, 16–25. 10.1111/nyas.12683 25773612 PMC4794983

[B8] Gordon R. L. Shivers C. M. Wieland E. A. Kotz S. A. Yoder P. J. McAuley J. D. (2015b). Musical rhythm discrimination explains individual differences in grammar skills in children. Dev. Sci. 18, 635–644. 10.1111/desc.12230 25195623

[B9] Grahn J. A. (2012a). Neural mechanisms of rhythm perception: current findings and future perspectives. Top. Cogn. Sci. 4, 585–606. 10.1111/j.1756-8765.2012.01213.x 22811317

[B10] Grahn J. A. (2012b). See what i hear? Beat perception in auditory and visual rhythms. Exp. Brain Res. 220, 56–61. 10.1007/s00221-012-3114-8 22623092

[B11] Hafter E. R. Sarampalis A. Loui P. (2008). Auditory attention and filters in Auditory Perception of Sound Sources, eds Yost W. A. Popper A. N. Fay R. R. (New York, NY: Springer), 115–142. 10.1007/978-0-387-71305-2_5

[B12] Halwani G. F. Loui P. Rüeber T. Schlaug G. (2011). Effects of practice and experience on the arcuate fasciculus: comparing singers, instrumentalists, and non-musicians. Front. Psychol. 2:156. 10.3389/fpsyg.2011.00156 21779271 PMC3133864

[B13] Henry M. J. Herrmann B. Obleser J. (2015). Selective attention to temporal features on nested time scales. Cereb. Cortex 25, 450–459. 10.1093/cercor/bht240 23978652

[B14] Herdener M. Humbel T. Esposito F. Habermeyer B. Cattapan-Ludewig K. Seifritz E. (2014). Jazz drummers recruit language-specific areas for the processing of rhythmic structure. Cereb. Cortex 24, 836–843. 10.1093/cercor/bhs367 23183709

[B15] Hoch L. Poulin-Charronnat B. Tillmann B. (2011). The influence of task-irrelevant music on language processing: syntactic and semantic structures. Front. Psychol. 2:112. 10.3389/fpsyg.2011.00112 21713122 PMC3112335

[B16] Jones M. R. Moynihan H. MacKenzie N. Puente J. (2002). Temporal aspects of stimulus-driven attending in dynamic arrays. Psychol. Sci. 13, 313–319. 10.1111/1467-9280.00458 12137133

[B17] Koelsch S. Gunter T. C. Wittfoth M. Sammler D. (2005). Interaction between syntax processing in language and in music: an ERP study. J. Cogn. Neurosci. 17, 1565–1577. 10.1162/089892905774597290 16269097

[B18] Kraus N. Chandrasekaran B. (2010). Music training for the development of auditory skills. Nat. Rev. Neurosci. 11, 599–605. 10.1038/nrn2882 20648064

[B19] Landau A. N. Fries P. (2012). Attention samples stimuli rhythmically. Curr. Biol. 22, 1000–1004. 10.1016/j.cub.2012.03.054 22633805

[B20] Large E. W. Jones M. R. (1999). The dynamics of attending: how people track time-varying events. Psychol. Rev. 106:119 10.1037/0033-295X.106.1.119

[B21] Large E. W. Snyder J. S. (2009). Pulse and meter as neural resonance. Ann. N. Y. Acad. Sci. 1169, 46–57. 10.1111/j.1749-6632.2009.04550.x 19673754

[B22] Lerdahl F. Jackendoff R. (1983). A Generative Theory of Tonal Music. Cambridge, MA: MIT Press.

[B23] Loui P. Kroog K. Zuk J. Winner E. Schlaug G. (2011). Relating pitch awareness to phonemic awareness in children: implications for tone-deafness and dyslexia. Front. Psychol. 2:111. 10.3389/fpsyg.2011.00111 21687467 PMC3108552

[B24] Loui P. Wessel D. L. Hudson Kam C. L. (2010). Humans rapidly learn grammatical structure in a new musical scale. Music Percept. 27, 377–388. 10.1525/mp.2010.27.5.377 20740059 PMC2927013

[B25] Mithen S. J. (2006). The Singing Neanderthals: The origins of Music: Language, Mind and Body. Cambridge, MA: Harvard University Press.

[B26] Patel A. D. (2003). Language, music, syntax and the brain. Nat. Neurosci. 6, 674–681. 10.1038/nn1082 12830158

[B27] Peretz I. Coltheart M. (2003). Modularity of music processing. Nat. Neurosci. 6, 688–691. 10.1038/nn1083 12830160

[B28] Perruchet P. Poulin-Charronnat B. (2013). Challenging prior evidence for a shared syntactic processor for language and music. Psychon. Bull. Rev. 20, 310–317. 10.3758/s13423-012-0344-5 23180417

[B29] Prinzmetal W. McCool C. Park S. (2005). Attention: reaction time and accuracy reveal different mechanisms. J. Exp. Psychol. Gen. 134, 73–92. 10.1037/0096-3445.134.1.73 15702964

[B30] Prinzmetal W. Zvinyatskovskiy A. Gutierrez P. Dilem L. (2009). Voluntary and involuntary attention have different consequences: the effect of perceptual difficulty. Q. J. Exp. Psychol. 62, 352–369. 10.1080/17470210801954892 18609402

[B31] Przybylski L. Bedoin N. Krifi-Papoz S. Herbillon V. Roch D. Léculier L.. (2013). Rhythmic auditory stimulation influences syntactic processing in children with developmental language disorders. Neuropsychology 27, 121–131. 10.1037/a0031277 23356600

[B32] Rohrmeier M. Rebuschat P. Cross I. (2011). Incidental and online learning of melodic structure. Conscious. Cogn. 20, 214–222. 10.1016/j.concog.2010.07.004 20832338

[B33] Schmidt-Kassow M. Kotz S. A. (2008). Entrainment of syntactic processing? ERP-responses to predictable time intervals during syntactic reanalysis. Brain Res. 1226, 144–155. 10.1016/j.brainres.2008.06.017 18598675

[B34] Schmidt-Kassow M. Kotz S. A. (2009). Event-related brain potentials suggest a late interaction of meter and syntax in the P600. J. Cogn. Neurosci. 21, 1693–1708. 10.1162/jocn.2008.21153 18855546

[B35] Slevc L. R. Okada B. M. (2015). Processing structure in language and music: a case for shared reliance on cognitive control. Psychon. Bull. Rev. 22, 637–652. 10.3758/s13423-014-0712-4 25092390

[B36] Slevc L. R. Rosenberg J. C. Patel A. D. (2009). Making psycholinguistics musical: self-paced reading time evidence for shared processing of linguistic and musical syntax. Psychon. Bull. Rev. 16, 374–381. 10.3758/16.2.374 19293110 PMC2658747

[B37] Sluming V. Barrick T. Howard M. Cezayirli E. Mayes A. Roberts N. (2002). Voxel-based morphometry reveals increased gray matter density in broca's area in male symphony orchestra musicians. Neuroimage 17, 1613–1622. 10.1006/nimg.2002.1288 12414299

[B38] Steinbeis N. Koelsch S. (2008). Shared neural resources between music and language indicate semantic processing of musical tension-resolution patterns. Cereb. Cortex 18, 1169–1178. 10.1093/cercor/bhm149 17720685

[B39] Trueswell J. C. Tanenhaus M. K. Kello C. (1993). Verb-specific constraints in sentence processing: separating effects of lexical preference from garden-paths. J. Exp. Psychol. Learn. Mem. Cogn. 19, 528–553. 10.1037/0278-7393.19.3.528 8501429

[B40] Zicarelli D. D. (1998). An extensible real-time signal processing environment for MAX, Paper Presented at the Proceedings of the International Computer Music Conference, (Ann Arbor, MI: University of Michigan).

