# Supplementary material for: Rhythmic Effects of Syntax Processing in Music and Language
Source: Front Psychol. 2015 Nov 23;6:1762. doi: 10.3389/fpsyg.2015.01762 (PMC4655243; doi:10.3389/fpsyg.2015.01762)
Supplement: Supplementary file 1 [file Table1.DOCX]

**Appendix**

Jung, Sontag, Park, & Loui. Rhythmic Effects of Syntax in Music and Language.

Comprehension Question stimuli for Experiments 1 and 2 to be matched to musical and linguistic stimuli from Slevc, Rosenberg, and Patel (2009).

***Item Target_answer Question***

1 Y Did the recipe call for parsley?

2 Y Was the scientist wearing contact lenses?

3 Y Did the award go to the producer's brother?

4 Y Did the fan hear that the song would be played on the radio next week?

5 Y Did the attorney think the defendant was innocent?

6 Y Did the author with the glasses write the novel?

7 Y Was the solution in the binder?

8 Y Is carpentry marketable in the countryside?

9 Y The teacher found the answer before class?

10 Y Did the superhero promise to help the woman?

11 Y Was the witness to testify for the defense?

12 Y Did the professor agree with the solution?

13 Y Did the boy see the giraffe today?

14 Y Was the diver an amateur?

15 Y Was the client going to get a cavity filled?

16 Y Did the neighbor warn the mailman?

17 Y Did the vet recommend medicine to cure an illness?

18 Y Did the child like to make snowmen at night?

19 Y Was the prince under a spell?

20 Y Did the man give his girlfriend a flower?

21 Y Does the vampire turn into a bat at dusk?

22 Y Did the stylist recommend conditioner?

23 Y Did the farmer go to the barn to feed the cows?

24 Y Was the student a boy?

25 Y Was the student writing for an essay?

26 Y Did the waitress spill coffee on purpose?

27 Y Did the students want the class to continue?

28 Y Did the clowns all drive to the circus in a Volkswagen Beetle?

29 Y Was the farm flooded?

30 Y Were the kids passing by the parade?

31 Y Did the guard see the prisoner escape?

32 Y Did the baby cry at a concert?

33 Y Was the football game exciting?

34 Y Did the guy order more food than he wanted?

35 Y Was the document backed up?

36 Y Was it overcast?

37 Y Did the manager spit before the pitch?

38 Y Did the judge determine the verdict?

39 Y Did the critic enjoy the movie?

40 Y Was the audience amused?

41 Y Did the boy catch the ball?

42 Y Were the kids eating bananas?

43 Y Did the children make the old man sad?

44 Y Did the student avoid the test?

45 Y Was the family's vacation disrupted by construction?

46 Y Did the students care about the material?

47 Y Did he go shopping with his girlfriend?

48 Y Did some of the employees stay home on Monday?

1 N Did the recipe call for basil?

2 N Was the scientist wearing glasses?

3 N Did the award go to the director's brother?

4 N Did the fan hear that the song would be played at the show next week?

5 N Did the attorney think the defendant was guilty?

6 N Did the author with the glasses write about the novel?

7 N Was the solution in the book?

8 N Is carpentry marketable in the city?

9 N The teacher found the answer after class?

10 N Did the superhero promise to stop the woman?

11 N Was the witness to testify for the prosecution?

12 N Did the professor disagree with the solution?

13 N Did the boy see the giraffe yesterday?

14 N Was this a professional diver?

15 N Did the client have an appointment?

16 N Did the boss warn the mailman?

17 N Did the vet tell the lady to administer the vaccine?

18 N Did the child like making snowmen?

19 N Was the princess under a spell?

20 N Were the flowers for the man's girlfriend?

21 N Can sunlight kill the vampire?

22 N Did the stylist think weekly shampooing was sufficient?

23 N Were the cows in the barn?

24 N Did the student take the final?

25 N Was the student writing for an exam?

26 N Did the waitress spill coffee by mistake?

27 N Did the students want the class end?

28 N Did the clowns all fit in the car?

29 N Was the farm dry?

30 N Did the kids enjoy the parade?

31 N Did the guard miss the prisoner's escape?

32 N Did the musician continue playing?

33 N Did the quarterback touch the ball?

34 N Did the guy order enough food?

35 N Did the computer cause trouble?

36 N Was the forecast wrong?

37 N Did the manager spit after the pitch?

38 N Did the jury have difficulty coming to a verdict?

39 N Did the critic dislike the movie?

40 N Was the audience surprised?

41 N Did the boy have friends?

42 N Were the monkeys fed?

43 N Did the old man care about his lawn?

44 N Was the student prepared for the test?

45 N Did the family leave the state?

46 N Did the students attempt to focus?

47 N Did the girlfriend miss the game?

48 N Did everyone go to work on Monday?
